# *Verruconis gallopava* pulmonary infection in a patient with Cushing’s syndrome

**DOI:** 10.1128/asmcr.00116-26

**Published:** 2026-10-13

**Authors:** Cole Schonhofer, Karol Boschung, Shaqil Peermohamed, John English, Claudine Desruisseaux, Marthe K. Charles, Suefay Liu

**Affiliations:** 1 Department of Pathology and Laboratory Medicine, University of British Columbia 8166, Vancouver, British Columbia, Canada; 2 Division of Infectious Diseases, Department of Medicine, University of British Columbia 8166, Vancouver, British Columbia, Canada; 3 Division of Medical Microbiology and Infection Control, Department of Pathology and Laboratory Medicine, Vancouver General Hospital, Vancouver Coastal Health 25469, Vancouver, British Columbia, Canada; Pattern Bioscience, Austin, Texas, USA

**Keywords:** *Verruconis gallopava*, dematiaceous mold, phaeohyphomycosis, invasive fungal infection, Cushing’s syndrome

## Abstract

**Background:**

*Verruconis gallopava* is a dematiaceous mold that rarely causes human infection except in immunocompromised patients, such as those with solid organ transplants or hematologic malignancies. We describe an unusual case of pulmonary *V. gallopava* infection in a patient with undiagnosed Cushing’s syndrome.

**Case Summary:**

A 48-year-old woman was admitted to the hospital for *Staphylococcus aureus* bacteremia, along with a history of recurrent falls and uncontrolled atrial fibrillation. As part of her investigations, she was noted to have an incidental lung nodule that was progressing in size, with evidence of cavitation over a 3-month period. A transbronchial biopsy revealed histopathologic findings consistent with phaeohyphomycosis, and bronchoscopy specimens grew *V. gallopava*. Fungal identification was confirmed via internal transcribed spacer and D1/D2 sequencing, as well as with matrix-assisted laser desorption/ionization time-of-flight mass spectrometry. After further investigation, the patient was diagnosed with Cushing’s syndrome secondary to an adrenal secretory mass and underwent left adrenalectomy, with subsequent clinical improvement. Repeat imaging following adrenalectomy showed radiographic resolution of her pulmonary nodule without initiation of antifungal treatment, consistent with clearance following immune reconstitution.

**Conclusion:**

This is the first reported case of invasive *V. gallopava* infection that self-resolved following immune reconstitution and without the need for antifungal therapy. *V. gallopava* is a rare cause of invasive fungal disease in immunocompetent patients, and workup for possible underlying immunodeficiency should be considered in patients with *V. gallopava* infection.

## INTRODUCTION

*Verruconis gallopava* is a dematiaceous mold that is a rare cause of human infection (1). Previously known as *Diplorhinotrichum gallopavum*, *Dactylaria gallopava*, *Ochroconis gallopava*, and *Dactylaria constricta*, *V. gallopava* is a thermophilic, saprophytic mold with septate pigmented hyphae, rhexolytic conidia in pairs, and production of a red diffusible pigment (2, 3). Originally described as an avian neurotropic pathogen, the first human cases were described in 1986 after isolation from bronchial washing fluid and subcutaneous abscesses (4, 5).

The majority of *V. gallopava* infections have been reported in immunocompromised individuals, predominantly solid organ transplant patients receiving immunosuppressing medications (1, 6, 7). Infection typically occurs in the lungs or skin with potential for brain dissemination, although there are reports of isolated brain infection as well. Reports of infection in immunocompetent patients are rare, as are reports in patients with non-transplant-related immunosuppression, such as chronic glucocorticoid exposure. Here we present a case of *V. gallopava* pulmonary infection in a patient ultimately diagnosed with Cushing’s syndrome.

## CASE PRESENTATION

A 48-year-old woman presented to the hospital with weakness, arthralgias, and recurrent falls. Her medical history was significant for atrial fibrillation, heart failure with preserved ejection fraction, and hypertension. Three months previously, while undergoing a cardiac computed tomography (CT) scan to assess for coronary artery disease, she was incidentally noted to have a 6 × 10 mm nodular opacity in the right middle lung lobe with surrounding ground-glass opacities and associated bronchial wall thickening with mucous plugging. At that time, she denied respiratory symptoms or significant mold, endemic fungi, or tuberculosis exposures, and a plan was made for interval follow-up.

On presentation, the patient was initially hypotensive with a blood pressure of 75/50, which resolved after a fluid bolus. She was otherwise vitally stable with a heart rate of 95 beats per minute and an oxygen saturation status of 98% on room air. She was afebrile with no other infectious symptoms. Multiple ecchymoses were noted in the bilateral lower and upper extremities, presumed secondary to bruising related to her recurrent falls, and her right ankle was also tender and swollen. Laboratory investigations are summarized in Table 1. Her blood cultures returned positive for methicillin-susceptible *Staphylococcus aureus* (MSSA) in four out of four bottles, and she was started on intravenous cefazolin. Repeat blood cultures at 48 hours were negative, and transthoracic echocardiogram showed no evidence of endocarditis. The source of her MSSA bacteremia was thought to be cutaneous trauma from recurrent falls. MSSA was also cultured from a purulent wound of the right ankle during her hospital admission.

**TABLE 1:** Laboratory investigations taken on admission as well as pertinent rheumatologic and immunologic test results throughout hospital admission^*a*^

Laboratory test	Result	Normal range
On admission		
Hemoglobin	84	120–155 g/L
White blood cell count	10.4	4.0–11.0 × 10^9^/L
Platelets	214	150–400 × 10^9^/L
Creatinine	135	50–90 µmol/L
C-reactive protein	142.5	<3.1 mg/L
Rheumatology		
ANA	Negative	Negative
ANCA	Negative	Negative
Rheumatoid factor	9	0–14 IU/mL
CCP	Negative	Negative
Complement C3 and C4	Negative	Negative
Immunology		
HIV EIA	Negative	Negative
Immunoglobulin G	5.2	5.8–15.6 g/L
Immunoglobulin A	1.42	0.70–4.00 g/L
Immunoglobulin M	0.75	0.50–3.00 g/L
Neutrophil oxidative burst test	Normal	Normal

^*a*^ ANA, antinuclear antibody; ANCA, antineutrophil cytoplasmic antibody; CCP, cyclic citrullinated peptide; EIA, enzyme immunoassay; HIV, human immunodeficiency virus.

**Fig 1:**
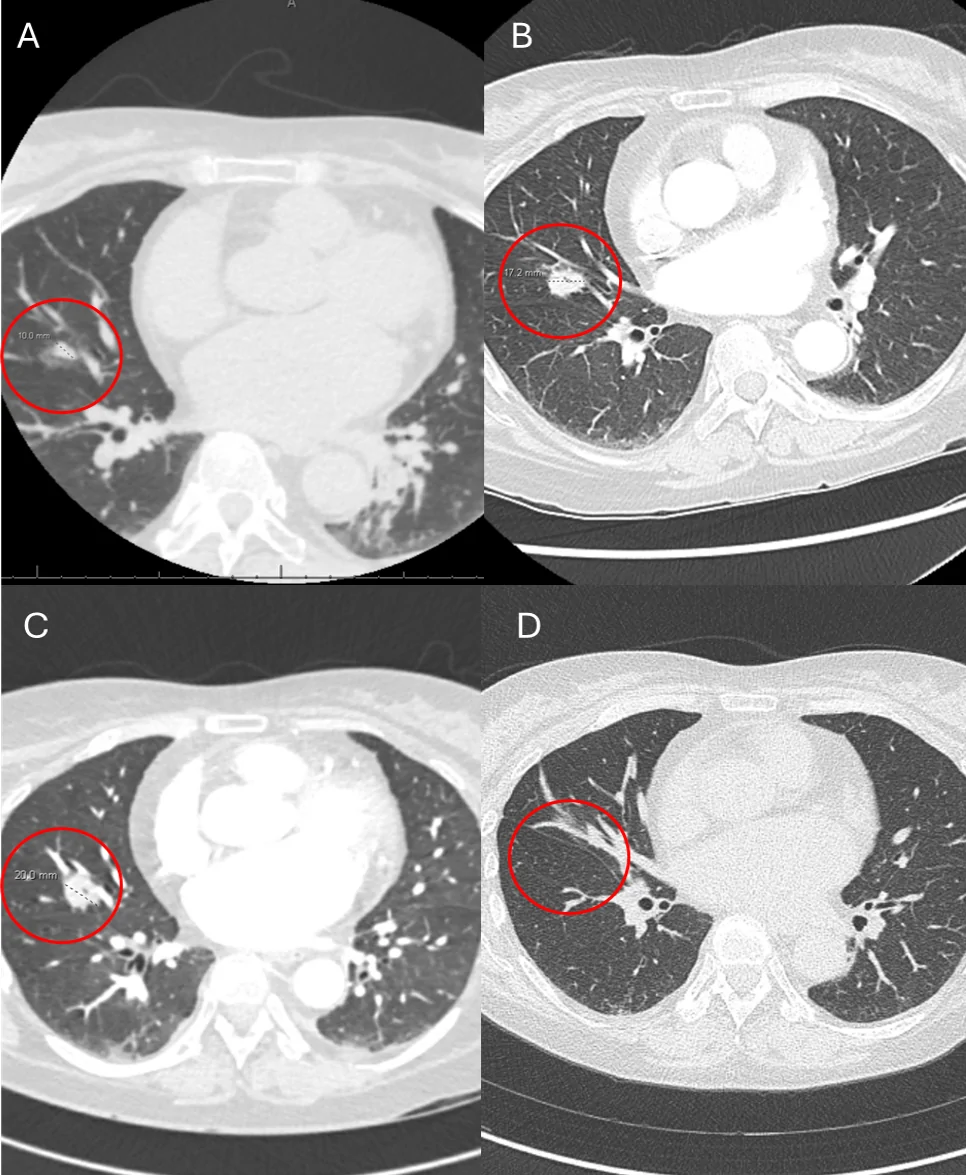
CT imaging of pulmonary nodule in the right middle lobe. (**A**) Initial presentation, 6–10 mm nodule with surrounding ground glass. (**B**) Three months later, 17 mm nodule with spiculation. (**C**) Three and a half months later, a 20 mm nodule with central lucency concerning for cavitation. (**D**) Interval resolution with residual scarring, 9 months from first appearance on imaging and 3 months after adrenalectomy.

**Fig 2:**
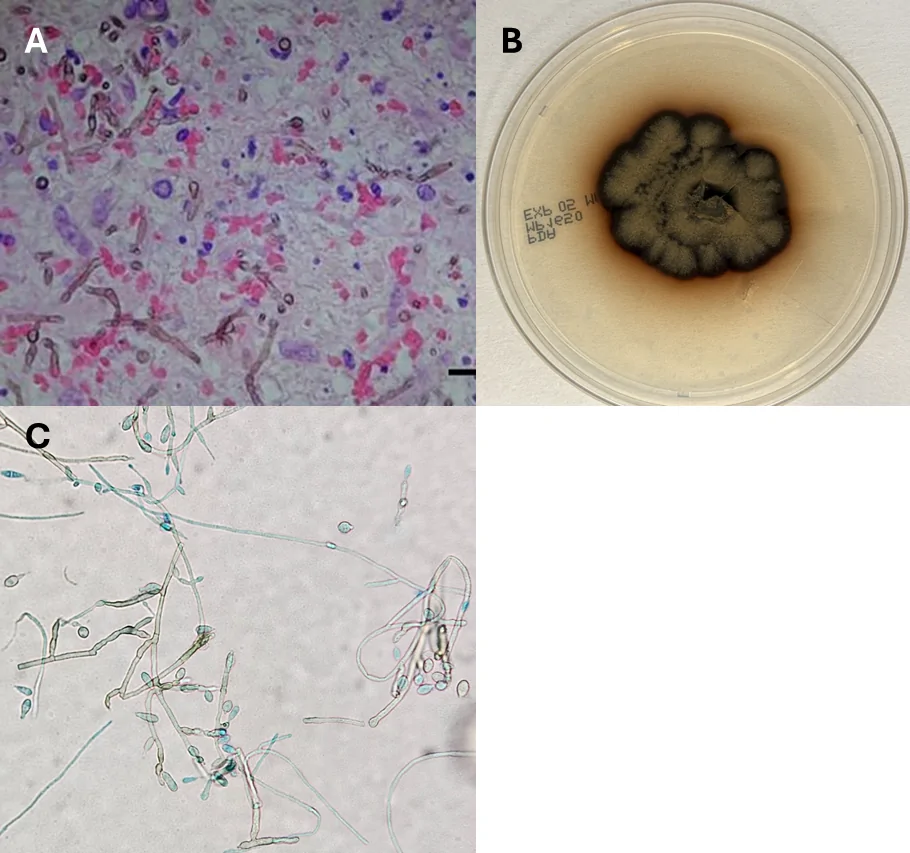
Histopathology and culture results from bronchoscopy. (**A**) Hematoxylin and eosin stain at 400× magnification demonstrating phaeohyphomycosis in pulmonary tissue acquired via EBUS-guided biopsy. (**B**) Appearance of felt-like pigmented colony with diffusible red pigment on potato-dextrose agar after 1 week of incubation at 28 ± 2°C. (**C**) Lactophenol cotton blue stain at 400× magnification, demonstrating septate pigmented hyphae and rhexolytic conidia.

CT of the chest demonstrated enlargement of her pulmonary nodule to 20 mm with a new central lucency suggestive of early cavitation (Fig. 1). Bronchoscopy with bronchoalveolar lavage (BAL) and endobronchial ultrasound (EBUS)-guided transbronchial and lymph node biopsy were performed with specimens sent for bacterial, mycobacterial, and fungal cultures, as well as histopathology. Histopathologic examination of the transbronchial biopsy sample demonstrated no evidence of malignancy or acid-fast bacilli. However, acute and chronic granulomatous inflammation was present, along with multiple pleomorphic fungal elements ranging in size from 1 to 7 µm (Fig. 2A). Ultimately, pathology findings were consistent with phaeohyphomycosis. Fungal culture from BAL was also positive after 4 days for a dematiaceous mold with a red diffusible pigment (Fig. 2B). Bacterial cultures grew only scant normal respiratory flora, and mycobacterial cultures were negative after 8 weeks of incubation.

The isolate was sent to the BC Centre for Disease Control Public Health Laboratory, where it was presumptively identified as *Verruconis gallopava* based on morphology and internal transcribed spacer (ITS) sequencing (no consensus sequence, but the forward sequence had 94.52% identity with 95% coverage, and the reverse sequence had 82% identity with 88% coverage). Identification confirmation via D1/D2 sequencing and antifungal susceptibility testing were performed at Alberta Precision Laboratories Public Health Laboratory. Antifungal susceptibilities revealed MIC values of 0.25 μg/mL for amphotericin B, 0.12 μg/mL for itraconazole, ≤0.015 µg/mL for micafungin, 0.06 μg/mL for posaconazole, and ≤0.03 µg/mL for voriconazole. Additionally, the isolate was correctly identified by matrix-assisted laser desorption/ionization time-of-flight (MALDI-TOF), via both the Bruker database (Bruker MBT Compass software version 5.1.410.9032 and BDAL Library version 12.0) with a score of 1.75B, as well as the Mass Spectrometry Identification (MSI)-2 (v2.0) database with a score of 21.03A.

Given the patient’s clinical improvement and lack of respiratory symptoms, antifungal treatment was deferred with a plan for thoracic surgery consultation regarding potential nodule excision. The patient was ultimately discharged and subsequently diagnosed with endogenous Cushing’s syndrome secondary to a secretory adrenal nodule for which she underwent a left adrenalectomy 2 months after her initial hospital admission. Repeat CT of the chest 4 months later revealed interval resolution of the pulmonary nodule with mild residual scarring. She never received antifungal therapy, and at her last clinic follow-up, she remained stable with no respiratory symptoms, along with improved cardiac function secondary to resolution of her Cushing’s syndrome.

## DISCUSSION

We report here a case of *V. gallopava* infection in a patient ultimately diagnosed with Cushing’s syndrome, with resolution following adrenalectomy. Although she had no respiratory symptoms, there was histopathologic evidence of phaeohyphomycosis from the EBUS-guided biopsy, consistent with proven infection as per international consensus definitions (8). It is possible that she may have developed symptoms had her lung lesion not been incidentally discovered early and her underlying immunodeficiency not been treated.

While this patient presented with localized pulmonary infection, *V. gallopava* also has neuroinvasive tendencies, often with devastating clinical outcomes (1, 9). Successful treatment with amphotericin B, voriconazole, itraconazole, posaconazole, and combinations of amphotericin B and triazoles has been described (6, 10, 11). Generally, the recommended first-line antifungal for localized phaeohyphomycosis is voriconazole, and the isolate in our case would have likely responded to treatment based on the low MIC value (12).

Overall, *V. gallopava* infection is rare and predominantly occurs in transplant patients, but it has occasionally been reported in immunocompetent individuals. In a PubMed literature review (search term *Verruconis* OR *gallopava* OR *gallopavum*), we found 41 articles describing 50 cases of *V. gallopava* infection. Of these, only five involved immunocompetent patients. The remaining 45 cases involved patients with solid organ transplant (*n* = 31), hematologic malignancy (*n* = 6), chronic glucocorticoid use (*n* = 4), hematopoietic stem cell transplant (*n* = 2), primary immunodeficiency (*n* = 1), or advanced HIV (*n* = 1).

Fungal isolates were identified via ITS and/or D1/D2 sequencing in 14 cases, while the remainder were identified via morphology and phenotype, or the identification method was not explicitly stated. No previously published cases describe the use of MALDI-TOF for identification of *V. gallopava*. Traditionally, identification of filamentous fungi via MALDI-TOF has been technically challenging; however, advances in extraction techniques and database updates have increased accuracy (13–16). In this case, extraction was done using the manufacturer-recommended mycelium transfer procedure, which is a direct-on-plate extraction process (16). While ITS and D1/D2 sequencing can require sample transfer to reference laboratories and feature prolonged turnaround times, MALDI-TOF can provide rapid same-day identification. In our case, MALDI-TOF accurately identified *V. gallopava* using both a commercial Bruker database and the MSI-2 database.

Interestingly, while we identified four previous reports of *V. gallopava* infection in patients on chronic, high-dose steroid therapy, we believe our report is the first to describe infection in a patient with endogenous glucocorticoid overproduction. Additionally, no published cases featured resolution without antifungal treatment. We found only one other published report of a patient who did not receive any antifungal treatment, in a patient with chronic lymphocytic leukemia who died from neuroinvasive infection and was diagnosed postmortem (17).

Immune dysregulation in Cushing’s syndrome is complex, but resultant elevated glucocorticoid levels are associated with more frequent infections with generally worse outcomes, including higher hospitalization rates, longer admissions, and increased mortality (18, 19). These patients are also at higher risk of invasive fungal infections, although this is more common in patients with exogenous than endogenous Cushing’s syndrome (20, 21). While invasive fungal infections with *Pneumocystis jirovecii*, *Candida*, *Cryptococcus*, and *Aspergillus* species are more common, there are rare reports of phaeohyphomycosis with dematiaceous fungi such as *Scedosporium*, *Alternaria*, *Exophiala*, and *Cladosporium* species (22–25). Following treatment of Cushing’s syndrome, glucocorticoid levels fall rapidly while full recovery of the hypothalamus-pituitary-adrenal axis typically occurs within several months (26). However, the exact timeline of functional immune reconstitution following treatment is unclear (27, 28). Given the lack of antifungal use and the timeline of our patient’s imaging resolution following definitive treatment of their Cushing’s syndrome, the patient’s immune reconstitution may have led to the cure of her localized phaeohyphomycosis.

### Conclusion

*V. gallopava* infection is rare and mainly affects immunosuppressed patients. When infection with *V. gallopava* is diagnosed in an apparently immunocompetent patient, clinicians should consider a thorough workup to rule out underlying immunodeficiency. If a treatable cause of immunodeficiency is found and addressed, localized infections may clear without antifungal therapy. Finally, MALDI-TOF can provide a rapid and accurate identification of *V. gallopava* cultured from clinical specimens.

## References

[1] Douglas AP, Chen SC-A, Slavin MA. 2016. Emerging infections caused by non-*Aspergillus* filamentous fungi. Clin Microbiol Infect 22:670–680. doi:10.1016/j.cmi.2016.01.011 26812445

[2] Samerpitak K, Van der Linde E, Choi H-J, Gerrits van den Ende AHG, Machouart M, Gueidan C, de Hoog GS. 2014. Taxonomy of *Ochroconis*, genus including opportunistic pathogens on humans and animals. Fungal Divers 65:89–126. doi:10.1007/s13225-013-0253-6

[3] Dixon DM, Polak-Wyss A. 1991. The medically important dematiaceous fungi and their identification. Mycoses 34:1–18. doi:10.1111/j.1439-0507.1991.tb00613.x 1922184

[4] Dixon DM, Salkin IF. 1986. Morphologic and physiologic studies of three dematiaceous pathogens. J Clin Microbiol 24:12–15. doi:10.1128/jcm.24.1.12-15.1986 PMC268822 3722358

[5] Fukushiro R, Udagawa S, Kawashima Y, Kawamura Y. 1986. Subcutaneous abscesses caused by *Ochroconis gallopavum*. Med Mycol 24:175–182. doi:10.1080/02681218680000271 3461148

[6] Terracol L, Hamane S, Euzen V, Denis B, Bretagne S, Dellière S. 2024. Phaeohyphomycosis due to *Verruconis gallopava*: rare indolent pulmonary infection or severe cerebral fungal disease? Mycopathologia 189:99. doi:10.1007/s11046-024-00903-9 39565406

[7] Giraldo A, Sutton DA, Samerpitak K, de Hoog GS, Wiederhold NP, Guarro J, Gené J. 2014. Occurrence of *Ochroconis* and *Verruconis* species in clinical specimens from the United States. J Clin Microbiol 52:4189–4201. doi:10.1128/JCM.02027-14 PMC4313285 25232157

[8] Donnelly JP, Chen SC, Kauffman CA, Steinbach WJ, Baddley JW, Verweij PE, Clancy CJ, Wingard JR, Lockhart SR, Groll AH, et al.. 2020. Revision and update of the consensus definitions of invasive fungal disease from the European organization for research and treatment of cancer and the mycoses study group education and research consortium. Clin Infect Dis 71:1367–1376. doi:10.1093/cid/ciz1008 PMC7486838 31802125

[9] Sides EH, Benson JD, Padhye AA. 1991. Phaeohyphomycotic brain abscess due to *Ochroconis gallopavum* in a patient with malignant lymphoma of a large cell type. J Med Vet Mycol 29:317–322. doi:10.1080/02681219180000481 1955951

[10] Xu L, Tao L. 2025. Pulmonary *Verruconis* infection in an immunocompetent patient: a case report and literature review. J Fungi (Basel) 11:634. doi:10.3390/jof11090634 PMC12471020 41003180

[11] Sharma D, Curry SR, Harris CE, Mills A, Burgoon R, Charles DW. 2025. *Verruconis gallopava*: an isavuconazonium foe. ASM Case Rep 1. doi:10.1128/asmcr.00081-25 PMC12530243 41244978

[12] Hoenigl M, Salmanton-García J, Walsh TJ, Nucci M, Neoh CF, Jenks JD, Lackner M, Sprute R, Al-Hatmi AMS, Bassetti M, et al.. 2021. Global guideline for the diagnosis and management of rare mould infections: an initiative of the European confederation of medical mycology in cooperation with the international society for human and animal mycology and the American Society for Microbiology. Lancet Infect Dis 21:e246–e257. doi:10.1016/S1473-3099(20)30784-2 33606997

[13] Trivitt GE, Lau AF. 2023. Performance of the MSI-2 database for fungal identification by matrix-assisted laser desorption ionization-time of flight mass spectrometry from cleanroom environments. J Clin Microbiol 61:e0135322. doi:10.1128/jcm.01353-22 PMC10035329 36853008

[14] Dogan Ö, Ozmen-Capin BB, White J, Serra C, Ahmed S, Botero-Kleiven S, Özenci V. 2026. Optimizing filamentous fungi identification by MALDI-TOF MS: a comparative analysis of key factors. Eur J Clin Microbiol Infect Dis 45:395–402. doi:10.1007/s10096-025-05316-0 PMC12987864 41145750

[15] Robert M-G, Cornet M, Hennebique A, Rasamoelina T, Caspar Y, Pondérand L, Bidart M, Durand H, Jacquet M, Garnaud C, Maubon D. 2021. MALDI-TOF MS in a medical mycology laboratory: on stage and backstage. Microorganisms 9:1283. doi:10.3390/microorganisms9061283 PMC8231132 34204665

[16] Fissel JA, Holdren-Serrell CK, Memon W, Zhang SX. 2022. Performance of a matrix-assisted laser desorption/ionization time-of-flight mass spectrometry testing algorithm for the rapid identification of clinical filamentous molds. Front Cell Infect Microbiol 12:915049. doi:10.3389/fcimb.2022.915049 PMC9240618 35782139

[17] Terreni AA, DiSalvo AF, Baker AS Jr, Crymes WB, Morris PR, Dowda H Jr. 1990. Disseminated *Dactylaria gallopava* infection in a diabetic patient with chronic lymphocytic leukemia of the T-cell type. Am J Clin Pathol 94:104–107. doi:10.1093/ajcp/94.1.104 2360556

[18] Hasenmajer V, Sbardella E, Sciarra F, Minnetti M, Isidori AM, Venneri MA. 2020. The immune system in Cushing’s syndrome. Trends in Endocrinology & Metabolism 31:655–669. doi:10.1016/j.tem.2020.04.004 32387195

[19] Minnetti M, Hasenmajer V, Sbardella E, Angelini F, Simeoli C, Di Paola N, Cozzolino A, Pivonello C, De Alcubierre D, Chiloiro S, Baldelli R, De Marinis L, Pivonello R, Pofi R, Isidori AM. 2022. Susceptibility and characteristics of infections in patients with glucocorticoid excess or insufficiency: the ICARO tool. Eur J Endocrinol 187:719–731. doi:10.1530/EJE-22-0454 PMC9641788 36102827

[20] Lionakis MS, Kontoyiannis DP. 2003. Glucocorticoids and invasive fungal infections. Lancet 362:1828–1838. doi:10.1016/S0140-6736(03)14904-5 14654323

[21] Scheffel RS, Dora JM, Weinert LS, Aquino V, Maia AL, Canani LH, Goldani LZ. 2010. Invasive fungal infections in endogenous Cushing’s syndrome. Infect Dis Rep 2:e4. doi:10.4081/idr.2010.e4 PMC3892578 24470886

[22] Chen Y-T, Lin H-C, Huang C-C, Lo Y-H. 2006. Cutaneous phaeohyphomycosis caused by an Itraconazole and Amphoterecin B resistant strain of *Veronaeae botryosa*. Int J Dermatol 45:429–432. doi:10.1111/j.1365-4632.2006.02619.x 16650171

[23] Bolland MJ, Bagg W, Thomas MG, Lucas JA, Ticehurst R, Black PN. 2004. Cushing’s syndrome due to interaction between inhaled corticosteroids and itraconazole. Ann Pharmacother 38:46–49. doi:10.1345/aph.1D222 14742792

[24] Hague J, Liotta E. 2003. Subcutaneous phaeohyphomycosis caused by *Exophiala jeanselmei* in an immunocompromised host. Cutis 72:132–134. 12953937

[25] Lee JY, Hyun M, Kim HA, Ryu SY. 2019. Unusual presentation of subcutaneous phaeohyphomycosis by *Alternaria alternate*. Ann Dermatol 31:563–566. doi:10.5021/ad.2019.31.5.563 PMC7992571 33911650

[26] Hurtado MD, Cortes T, Natt N, Young WF, Bancos I. 2018. Extensive clinical experience: hypothalamic-pituitary-adrenal axis recovery after adrenalectomy for corticotropin-independent cortisol excess. Clin Endocrinol (Oxf) 89:721–733. doi:10.1111/cen.13803 PMC6246804 29968420

[27] Vogel F, Braun L, Zopp S, Nowak E, Schreiner J, Benz I, Rubinstein G, Künzel H, Ritzel K, Kroiss M, Honegger J, Beuschlein F, Schilbach K, Teupser D, Bidlingmaier M, Reincke M. 2023. Low-grade inflammation during the glucocorticoid withdrawal phase in patients with Cushing’s syndrome. Eur J Endocrinol 188:375–384. doi:10.1093/ejendo/lvad041 36971260

[28] Pivonello R, Isidori AM, De Martino MC, Newell-Price J, Biller BMK, Colao A. 2016. Complications of Cushing’s syndrome: state of the art. Lancet Diabetes Endocrinol 4:611–629. doi:10.1016/S2213-8587(16)00086-3 27177728

